# Risk factors for perioperative venous thromboembolism: A retrospective study in Japanese women with gynecologic diseases

**DOI:** 10.1186/1477-9560-8-17

**Published:** 2010-11-07

**Authors:** Nao Suzuki, Norihito Yoshioka, Tatsuru Ohara, Noriyuki Yokomichi, Takafumi Nako, Namiko Yahagi, Suguru Igarashi, Yoichi Kobayashi, Misako Yoshimatsu, Kenji Takizawa, Yasuo Nakajima, Kazushige Kiguchi, Bunpei Ishizuka

**Affiliations:** 1 Department of Obstetrics and Gynecology, St. Marianna University School of Medicine, Kanagawa, Japan; 2 Department of Radiology, St. Marianna University School of Medicine, Kanagawa, Japan

## Abstract

**Background:**

Patients with gynecologic cancer have a high risk of venous thromboembolism (VTE) like patients with other cancers. However, there is little information on risk factors for VTE during gynecologic surgery and no uniform preventive strategy. Our objectives were to identify risk factors for perioperative VTE in gynecologic patients and establish methods for prevention.

**Methods:**

We analyzed 1,232 patients who underwent surgery at the Department of Obstetrics and Gynecology of St. Marianna University School of Medicine between January 2005 and June 2008. We investigated (1) risk factors for preoperative VTE, (2) use of an inferior vena cava (IVC) filter, and (3) risk factors for postoperative VTE.

**Results:**

There were 39 confirmed cases of perioperative VTE (3.17%), including 25 patients with preoperative VTE and 14 with postoperative VTE. Thirty-two patients had cancer and seven patients had benign diseases. Twenty-two of the 32 cancer patients (68.7%) had preoperative VTE, while postoperative VTE occurred in 10 cancer patients. Multivariate analysis indicated that ovarian cancer, tumor diameter ≥10 cm, and previous of VTE were independent risk factors for preoperative VTE. Among ovarian cancer patients, multivariate analysis showed that an age ≥50 years, the presence of heart disease, clear cell adenocarcinoma, and tumor diameter ≥20 cm were independent risk factors for preoperative VTE. The factors significantly related to preoperative VTE in patients with benign disease included previous VTE, age ≥55 years, tumor diameter ≥20 cm, and a history of allergic-immunologic disease. Thirteen of the 25 patients (52%) with preoperative VTE had an IVC filter inserted preoperatively. Postoperative screening (interview and D-dimer measurement) revealed VTE in 14/1,232 patients (1.14%). Multivariate analysis indicated that cancer surgery, a history of allergic-immunologic disease, and blood transfusion ≥2,000 ml were independent risk factors for postoperative VTE.

**Conclusions:**

Perioperative VTE is often fatal and preventive measures should be taken in the gynecologic field, especially when patients have the risk factors identified in this study. Since VTE is often present before surgery, preoperative screening is important and use of an IVC filter should be considered.

## Background

In recent years, a rapid increase of venous thromboembolism (VTE) has occurred in Japan with aging of the population, more complex operations, and new therapeutic procedures related to improvements of catheterization and implantation techniques [1]. A previous study of patients undergoing abdominal surgery by Sakon et al. identified four factors, which were (1) female sex, (2) intrapelvic surgery, (3) an age ≥60 years, and (4) an operating time ≥3 hours, as risk factors for postoperative VTE [1]. The incidence of VTE increased along with the number of risk factors and exceeded 60% in patients with all four factors. The authors concluded that VTE is common in Japanese patients after major abdominal surgery and it is important to consider drug prophylaxis, especially in patients with multiple risk factors [1]. According to Edition 8 of the American College of Chest Physicians (ACCP) Guidelines, the incidence of asymptomatic deep venous thromboembolism (DVT) based on objective diagnostic screening is 15-40% among patients undergoing major gynecologic surgery without preventive measures, which is the same as for general surgery, major urological operations, and neurosurgery [2]. The results of a survey of perioperative pulmonary thromboembolism (PE) in Japan showed a prevalence of 4.41 episodes per 10,000 operations. The number of cases in the gynecology field ranked third behind orthopedic surgery and gastroenterology [3]. When VTE prophylaxis was not performed, the occurrence of fatal PE increased, the cost of treatment became higher, recurrence was more common, and major adverse events (such as the chronic thrombotic syndrome) occurred [2]. Preoperative and perioperative/postoperative PE accounted for 24.3% and 75.6% of these cases, respectively. Since almost all PE occurred postoperatively, especially after laparotomy for malignant tumors, both preoperative/perioperative prevention and prevention of postoperative VTE are important in such patients [3]. The above reports suggest that it is also necessary to strengthen preventive measures for perioperative VTE in the field of gynecology, and it is important to identify the risk factors for this condition.

Surgery for gynecologic cancer shows several differences from general surgery. Patients often require intrapelvic procedures, such as lymph node dissection and excision of peritoneal metastases, and it is likely that VTE will occur at a high incidence in these cases. Lymph node dissection is one of the risk factors for VTE because of possible venous damage [4]. In the gynecologic cancer field, 50% of patients with postoperative PE have endometrial cancer because 62.5% of such patients have a body mass index (BMI) ≥25 kg/m^2 ^and standard surgery includes lymph node dissection [4]. Dissection of para-aortic lymph nodes is also sometimes performed, which significantly increases the incidence of VTE compared to that in patients only receiving dissection of the intrapelvic nodes [4]. Because lymph node dissection increases the risk of thrombosis during or soon after surgery and residual tumor sometimes exists postoperatively in ovarian cancer patients, it is necessary to take preventive measures against VTE from the early postoperative period. It has been reported 45% of VTE occurs on the day of surgery [5], so it is important to use methods such as intermittent pneumatic compression (IPC) during and after surgery to prevent VTE.

According to an analysis of 1,974 patients with gynecologic cancer by Santoso et al., the incidence of DVT was 4.2% (36/853) and 0.2% (2/1,121) in patients with gynecological cancer or benign tumors, respectively, and the risk of DVT was 2.8 times higher in gynecologic cancer patients [6]. An analysis of 6,218 patients with gynecologic disease receiving IPC as the only preventive method showed an incidence of 0.68% (42/6,218) for postoperative PE, including 0.32% (10/3,518) for benign disease and 2.21% (32/1,451) for cancer patients [4]. Multivariate analysis of patients with and without IPC showed significant differences in the risk of PE for cancer surgery, BMI ≥25 kg/m^2^, and blood transfusion, with the risk ratios being 2.860, 3.922, and 3.834, respectively. Therefore, obese women receiving blood transfusion postoperatively are high-risk patients who require very strict management. With prophylactic IPC, the risk of postoperative PE was significantly reduced by about 60% (risk ratio: 0.396), indicating that at least employing IPC after gynecologic surgery is important [4]. According to a survey of 1,073 gynecologic oncologists from the Society of Gynecologic Oncology undertaken by Martino et al., 41% only performed IPC to prevent perioperative VTE, while 16% only used anticoagulant therapy and 42% used both methods [7]. At present, a preventive strategy has not been established for postoperative VTE in cancer patients, including those with gynecologic cancer [8]. In Japan, it is assumed that physicians are unlikely to perform both IPC and anticoagulant therapy at a higher level than in the above report. When we investigated this issue previously [4], prevention of postoperative VTE was limited to use of IPC and anticoagulation was not performed at that time. In the present study, we investigated risk factors related to postoperative VTE in patients with gynecologic disease who received both IPC and anticoagulant therapy. Among cancer patients, Camps et al. reported that ovarian cancer follows pancreatic, gastric, urological, and brain cancer as the disease with the highest risk of VTE [9]. Since it is therefore quite possible that VTE will have already occurred before the start of treatment, it is important to undertake preoperative screening of ovarian cancer patients. In the present study, therefore, we also investigated the risk factors related to preoperative VTE in patients with gynecologic diseases.

## Methods

We analyzed 1,232 patients who underwent surgery at the Department of Obstetrics and Gynecology of St. Marianna University School of Medicine (Kanagawa, Japan) between January 2005 and June 2008. Surgery for obstetric procedures or infertility and cervical conization were excluded. Patients were evaluated by measurement of the D-dimer level (Mitsubishi Chemical Iatron, Tokyo, Japan), as well as by electrocardiography, arterial blood gas analysis, chest X-ray examination, and echocardiography. Confirmation of the diagnosis of VTE was done by chest helical computed tomography and ultrasonography of leg vein pulsation. We performed the chest helical CT and ultrasonography of leg vein to the patients with high D-dimer levels before and after the operation. IPC alone or IPC combined with an anticoagulant [danaparoid sodium (Schering-Plough, Osaka, Japan) administered intravenously at 1,250 units/day for 5 to 7 days starting preoperatively] was employed to prevent postoperative VTE. As previously reported [4], the combination of IPC and anticoagulation (danaparoid sodium) was routinely used for cancer patients. Patients with benign disease and a BMI ≥25 kg/m^2 ^or those receiving blood transfusion were also given the combination of IPC and danaparoid sodium. This combined prophylaxis was performed in 23.1% (284/1,232) of all surgical patients.

### Statistical analysis

To investigate factors related to preoperative VTE, univariate analysis was performed to assess the relation between VTE and 14 variables, including the age (≥50 years old or ≥55 years old), BMI (≥25 kg/m^2 ^or ≥28 kg/m^2^), presence or absence of complications (hypertension, abnormal glucose tolerance, allergic-immunologic disease, heart disease, hyperlipidemia, and VTE), use or non-use of hormone preparations, benign or malignant disease, and tumor diameter (≥10 cm or ≥20 cm). Selection of variables was performed by the stepwise method or the scoring method. Then the following five variables were employed for multivariate analysis (Multiple Logistic Regression): age ≥50 years, presence/absence of heart disease, presence/absence of VTE, ovarian cancer, and tumor diameter ≥10 cm.

To assess factors related to preoperative VTE in ovarian cancer patients, univariate analysis was performed with 14 variables, which were the same as those listed above, except that "clear cell adenocarcinoma" was substituted for "benign or malignant disease". Variable selection was performed by the stepwise or scoring methods, resulting in selection of the following four variables for multivariate analysis (Multiple Logistic Regression): age ≥50 years, presence or absence of heart disease, clear cell adenocarcinoma, and tumor diameter ≥20 cm.

For investigation of factors related to preoperative VTE in patients with benign diseases, univariate analysis was performed with 13 variables after deleting "benign or malignant disease" from the initial 14 variables. To detect factors related to postoperative VTE, univariate analysis was done to assess the relation between VTE and the following 21 variables: age (≥50 years old or ≥55 years old), BMI (≥25 kg/m^2 ^or ≥28 kg/m^2^), presence or absence of complications (hypertension, abnormal glucose tolerance, allergic-immunologic disease, heart disease, hyperlipidemia, and VTE), use or non-use of hormone preparations, benign or malignant disease, operating time (≥4 hours or ≥6 hours), postoperative blood loss (≥1,000 ml, ≥1,500 ml, or ≥2,000 ml), perioperative blood transfusion (≥1,000 ml and ≥2,000 ml), and tumor diameter (≥10 cm or ≥20 cm). After variable selection was performed by the stepwise or scoring methods, the following three variables were used for multivariate analysis (Multiple Logistic Regression): the presence or absence of allergic-immunologic disease, surgery for benign or malignant disease, and perioperative blood transfusion ≥2,000 ml.

All statistical analyses were performed using SAS software version 8.2 (SAS Institute, Cary, NC, USA).

### Indications for inferior vena cava (IVC) filter insertion

The D-dimer level was measured and computed tomography (CT) was performed immediately before surgery in cancer patients and patients with large benign tumors. An IVC filter (Gunter Tulip Vena Cava Filter, Cook Medical) was inserted preoperatively if PE was detected and there was pulmonary dysfunction due to microthrombi or if proximal DVT was detected even in the absence of PE.

## Results and Discussion

### Characteristics of VTE patients with gynecologic diseases (Table 1)

We analyzed 1,232 patients who underwent surgery at the Department of Obstetrics and Gynecology of St. Marianna University School of Medicine between January 2005 and June 2008. VTE was diagnosed in 39/1,232 patients (3.17%), being preoperative in 25/39 patients (64.1%) and postoperative in 14 patients (35.9%). Thirty-two of 39 patients (82.1%) had cancer and seven (17.9%) had benign disease. Sixteen (64%) of the preoperative VTE patients already had PE, emphasizing the importance of screening for VTE and PE before surgery. Postoperative VTE occurred in 14 out of 39 patients (35.9%). According to previous reports, the incidence of postoperative DVT and PE in gynecologic cancer patients was 7-45% and 1-2.6%, respectively [10-12]. Cancer was present in 32 of the 39 VTE patients (82.1%) with gynecologic disease and perioperative VTE was significantly more common in cancer patients, as reported previously [13]. Among patients undergoing surgery for cancer, the incidence of VTE was 8.70% (32/368). Among the 32 cancer patients, the majority had preoperative VTE (68.7%; 22/32), while 10 patients had postoperative VTE. The incidence of VTE in patients with uterine cervical cancer, endometrial cancer, and ovarian cancer was 3%, 2.56%, and 18.1% respectively. The incidence of VTE was significantly higher in patients with ovarian cancer than in those with other cancers (*p *< 0.01, Student's *t*-test). Among patients with benign disease, only 7/864 (0.81%) had VTE, which was preoperative in three of the seven patients (42.9%) and postoperative in four patients (57.1%). These results indicated that perioperative VTE is significantly more common among gynecological patients with cancer (*p *< 0.01, Student's *t*-test). There were no significant differences of age and BMI between patients with preoperative or postoperative VTE, but tumor diameter was significantly larger in patients with a postoperative onset (*p *< 0.01, Student's *t*-test).

**Table 1:** Incidence and type of perioperative VTE in patients with gynecologic disease

	Total VTE (n)	Preoperative (n)	Postoperative (n)
All gynecologic diseases (n = 1,232)	39	25	14
Benign diseases (n = 864)	7	3	4
Malignant diseases (n = 368)	32	22	10
Uterine cervical cancer (n = 100)	3	0	3
Endometrial cancer (n = 117)	3	1	2
Ovarian cancer/tubal cancer (n = 144)	26	21	5
Age, years (range)		60.6 (42-88)	58.0 (40-75)
BMI, kg/m^2 ^(range)		21.7 (16.6-28.5)	22.2 (16.4-27.8)
Tumor diameter, cm (range)		16.9 (6-30)	10.3 (3-20)

*: Significant difference by Student's *t*-test (*p *< 0.01)

BMI: body mass index; VTE: venous thromboembolism.

### Characteristics of patients with gynecologic disease and preoperative VTE

Screening for preoperative VTE by interview, D-dimer testing, and preoperative imaging showed that only six out of 25 patients (24%) with preoperative VTE had symptoms such as dyspnea, while most of them had silent thrombosis. The mean D-dimer value of the 25 patients was 20.3 μg/ml and IVC filters were inserted preoperatively in 13 of them (52%).

VTE was present preoperatively in 22 out of 368 cancer patients (5.98%) and 21 of them (95.5%) had ovarian cancer or tubal cancer. Thus, in 14.6% (21/144) of the patients scheduled to undergo surgery for ovarian or tubal cancer, VTE was present preoperatively (Table 1).

There was DVT only, PE only, or DVT plus PE in nine, two, and 14 patients, respectively, and 16 of the 25 patients (64%) had PE.

### Results of multivariate analysis of preoperative VTE

Since an accurate screening method for perioperative VTE is not available at present, we investigated risk factors for preoperative VTE in patients undergoing gynecologic surgery by performing univariate and multivariate analyses. The initial univariate analysis identified age (≥50 years old and ≥55 years old), a history of VTE, ovarian cancer, and tumor diameter (≥10 cm and ≥20 cm) as being significantly related to preoperative VTE (Table 2). When multivariate analysis was performed with the five variables shown in Table 2, there was a significant association between VTE and ovarian cancer, tumor diameter ≥10 cm, and a history of VTE, with the risk ratios being 20.521, 6.442, and 34.596, respectively.

**Table 2:** Risk factors for preoperative VTE

Univariate analysis				
Variable		*p *value		

Age ≥50 years		<0.01		
Age ≥55 years		<0.01		
History of VTE		<0.01		
Ovarian cancer		<0.01		
Tumor diameter ≥10 cm		<0.01		
Tumor diameter ≥20 cm		<0.01		

**Multivariate analysis**				
Variable	Risk ratio	95% CI		*p *value

		Lower	Upper	
History of VTE	34.596	2.003	597.612	<0.05
Age ≥50 years	2.368	0.657	8.531	0.187
Ovarian cancer	20.521	5.046	83.455	<0.01
Tumor diameter ≥10 cm	6.442	1.280	32.412	<0.05
Heart disease	0.763	0.032	18.2	0.867

CI: confidence interval; VTE: venous thromboembolism.

Fotopoulou et al. reported an analysis of 2,743 patients with ovarian cancer from in three prospective randomized trials of postoperative platinum/paclitaxel-based chemotherapy performed by the Arbeitsgemeinschaft Gynaekologische Onkologie Ovarian Cancer Study Group [14]. They found 76 cases of VTE among patients who received 6 to 11 cycles of chemotherapy and it occurred within 2 months postoperatively in 50% of these patients. Their multivariate analysis showed that a BMI ≥30 kg/m^2 ^and old age were independent risk factors for VTE in ovarian cancer patients. The overall survival rate was significantly reduced by VTE and PE was a significant adverse prognostic factor [14]. According to an analysis of 13,301 ovarian cancer patients in the California Cancer Registry, 5.2% of the patients developed VTE within 2 years of diagnosis and it has an adverse effect on their prognosis. Certain coagulation-related factors produced by ovarian cancer cells may increase the risk of VTE and this can be decreased by 30% after the tumor size is reduced by surgery [15].

### Characteristics of preoperative VTE in ovarian cancer patients

The mean age and mean tumor diameter of the 20 ovarian cancer patients with preoperative VTE (excluding tubal cancer patients) was 58.2 (43-75) years and 18.7 (10-27) cm, respectively. Two patients (10%) had serous carcinoma, while the histology was endometrioid in five patients (25%), mucinous in one patient (5%), and clear cell in 12 patients (60%). Since we found that 21 out of 22 cancer patients (95.5%) with preoperative VTE had ovarian or tubal cancer, we performed an analysis of variables related to the occurrence of preoperative VTE in ovarian cancer patients. The results of univariate analysis using 14 variables showed that a diagnosis of clear cell adenocarcinoma and the tumor diameter (≥10 cm or ≥20 cm) were significantly associated with preoperative VTE (Table 3). When multivariate analysis was performed using four variables, a significant association with VTE was observed for a tumor diameter ≥20 cm, clear cell adenocarcinoma, and age ≥50 years, with risk ratios being 29.942, 9.052, and 9.953, respectively (Table 3).

**Table 3:** Risk factors for preoperative VTE in ovarian cancer patients

Univariate analysis				
Variable		*p *value		

Clear cell adenocarcinoma		<0.05		
Tumor diameter ≥10 cm		<0.05		
Tumor diameter ≥20 cm		<0.01		

**Multivariate analysis**				

Variable	Risk ratio	95% CI		*p *value
		Lower	Upper	

Tumor diameter ≥20 cm	29.942	5.402	165.946	<0.01
Clear cell adenocarcinoma	9.052	1.929	42.477	<0.01
Age ≥50 years	9.953	1.338	74.047	<0.05
Heart disease	2.442	0.088	67.533	0.598

CI: confidence interval; VTE: venous thromboembolism.

In support of our results, it has been reported that ovarian cancer is associated with the same high risk of VTE as pancreatic cancer and brain cancer [16]. Tateo et al. reported that ovarian cancer was associated with the highest risk of VTE among gynecologic diseases [17]. A study of 253 ovarian cancer patients revealed VTE in 42 patients (16.6%) and PE in four of these 42 patients (1.6%). VTE was discovered before the start of treatment in 3.2% (n = 8), postoperatively in 2.4% (n = 6), during first-line postoperative chemotherapy in 4.8% (n = 12), and during post-treatment follow-up in 4.8% (n = 12). Multivariate analysis indicated that independent risk factors for the occurrence of VTE during the treatment of ovarian cancer were the presence of residual tumor, age (risk increasing with each decade), and BMI (risk increasing with each 5 kg/m^2^). Unlike Tateo et al. [17], we investigated ovarian cancer patients with confirmed preoperative VTE, but both studies indicate that tumor diameter and age are important factors related to VTE. However, the role of clear cell carcinoma may be specific to Japan. Satoh et al. previously reported that clear cell carcinoma and massive ascites are risk factors for preoperative DVT in ovarian cancer patients based on multivariate analysis [18]. Satoh et al. also reported that extrauterine progression and histology other than endometrioid adenocarcinoma were risk factors for preoperative VTE in endometrial cancer patients [19]. Furthermore, Black et al. found that progressive ovarian cancer (stages III and IV), ascites, and residual lesions ≥1 cm independent risk factors for postoperative VTE in patients with ovarian or tubal cancer [20].

### Characteristics of preoperative VTE in patients with benign diseases

Among 864 patients with benign gynecological diseases, preoperative VTE was found in only three (0.35%). These three patients were an 80-year-old woman with an ovarian tumor measuring 23 cm in diameter and a history of VTE, a 42-year-old woman with an ovarian tumor and a history VTE, and an 83-year-old woman with a BMI of 24 kg/m^2^, hyperlipidemia, and hypertension. The mean age, mean BMI, and mean tumor diameter of the three patients were 68.3 (42-83) years, 21.1 (15.0-24.7) kg/m^2^, and 12 (6-23) cm, respectively. Univariate analysis of factors related to preoperative VTE in patients with benign disease using 13 variables showed that a history of VTE (*p *< 0.01), age ≥55 years (*p *< 0.01), tumor diameter ≥20 cm (*p *< 0.01), and history of allergic-immunologic disease (*p *< 0.05) were significant risk factors (Table 4). Multivariate analysis could not be performed because only three patients had preoperative VTE.

**Table 4:** Risk factors for preoperative VTE in benign disease patients (univariate analysis)

Variable	*p *value
History of VTE	<0.01
Age ≥55 years	<0.01
Tumor diameter ≥20 cm	<0.01
Allergic-immunologic disease	<0.05

VTE: venous thromboembolism.

Postoperative VTE was detected in four of the 864 patients with benign diseases (0.46%). These four patients had ovarian tumors (n = 2), uterine myoma (n = 1), and intrauterine infection (n = 1). The mean age, mean BMI, mean tumor diameter, mean operating time, and mean blood loss were 53.8 years, 23.0 kg/m^2^, 12 cm, 144.3 minutes, and 1,154 ml, respectively. There were no significant differences of these parameters between the 10 cancer patients and four benign disease patients with postoperative VTE. Santoso et al. reported that the incidence of postoperative DVT was 0.30% (2/720) in patients with benign disease [6]. Martino et al. reported that the incidence of PE after major surgery in patients with benign disease was 0.3% (1/332) [13], while DVT was detected in 22.2% (2/9) of symptomatic patients with benign disease. These results indicate that the prevalence of perioperative VTE among patients with benign gynecological diseases is significantly lower compared with that among cancer patients. However, even in patients with benign disease, screening for VTE should be performed preoperatively when patients have risk factors such as a history of VTE, age ≥55 years, tumor diameter ≥20 cm, or a history of allergic-immunologic disease, and postoperative screening should be done in patients who require a large volume of blood transfusion.

### Preoperative prevention

In our series, an IVC filter was inserted preoperatively in 13 out of 25 patients (52%) with preoperative VTE (Table 5). These 13 patients consisted of one with a benign ovarian tumor, one with endometrial cancer, and 12 with ovarian cancer. Both PE and DVT were present in nine patients, while one patient had PE alone and three patients had DVT alone. The filter was placed a mean of 3.62 (1-15) days preoperatively and was inserted 1-2 days preoperatively in the majority of cases (n = 8). The mean D-dimer level of the 13 patients was 20.4 (2.0-75.8) μg/ml. Four patients had the filter removed after 10 to 15 days.

**Table 5:** Patients with preoperative insertion of an IVC filter

Patient No.	Disease	PE	DVT	Timing of insertion (days before surgery)
1	Benign ovarian tumor	-	+	1 day
2	Endometrial cancer	+	+	7 days
3	Ovarian cancer	+	+	2 days
4	Ovarian cancer	-	+	2 days
5	Ovarian cancer	+	+	1 day
6	Ovarian cancer	+	+	5 days
7	Ovarian cancer	+	+	2 days
8	Ovarian cancer	+	+	1 day
9	Ovarian cancer	-	+	6 days
10	Ovarian cancer	+	+	1 day
11	Ovarian cancer	+	+	1 day
12	Ovarian cancer	+	-	3 days
13	Ovarian cancer	+	+	15 days

DVT: deep venous thromboembolism; IVC: inferior vena cava; PE: pulmonary thromboembolism.

**Figure 1:**
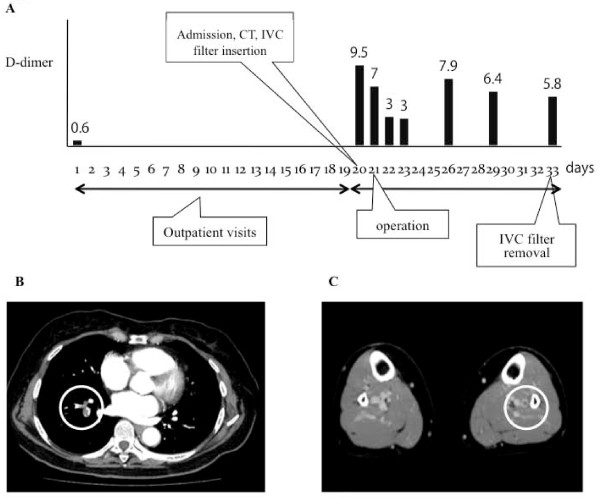
**Profile of D-dimer values and preoperative CT findings in patient number 5**. A: Day 1: first outpatient examination. Day 20: admission, CT and IVC filter insertion. Day 21: operation. Day 33: IVC filter removal. B: Superior and inferior lobe branches of the right pulmonary artery are not visualized (white circles). C: Posterior tibial vein and peroneal vein in the left leg show no enhancement (white circles).

Case number 5 was 67 years old, nulligravid and nulliparous, with menopause at 48 years of age. Her main complaint was an abdominal mass and ovarian cancer was suspected. She had a history of hypertension and a BMI of 25.9 kg/m^2^. The D-dimer value on the day of first examination (day 1) was 0.6 μg/ml. Since the D-dimer level was a high 9.5 μg/ml (Figure 1A) on the day of admission (day 20), contrast CT was performed on the same day, resulting in the diagnosis of PE and DVT (Figures 1B and 1C). On the days of the initial visit and admission, there were no symptoms or signs of VTE. After insertion of an IVC filter via the right subclavian vein on the day before surgery (day 20), staging laparotomy was performed (day 21). After confirming the resolution of VTE by CT, the filter was removed on day 13 after insertion (day 33). The histopathologic diagnosis was ovarian clear cell adenocarcinoma of International Federation of Gynecology and Obstetrics (FIGO) stage IIc. When VTE is detected by preoperative screening, surgery is often postponed because of anticoagulant therapy, etc. However, a retrospective study of IVC filters in 39 patients with gynecologic cancer by Adib et al. found no filter-related adverse events, indicating that it is better to insert a filter and not delay the start of treatment [21]. They reported that it was possible to perform surgery within 6 days after diagnosis of VTE in 17 of the 39 patients (43%). We inserted an IVC filter preoperatively in 13 out of 25 patients with preoperative VTE (52%), and no filter-related adverse events were observed. The filter was inserted a mean of 3.62 (1-15) days preoperatively and the majority of filters were inserted 1-2 days preoperatively (n = 8). Case number 5 with ovarian clear cell carcinoma did not have VTE at the initial outpatient assessment, but VTE was detected immediately before surgery. An IVC filter was inserted and radical surgery could be performed on the next day without postponing treatment. Eleven of our 13 patients (84.6%) with IVC filters had ovarian cancer. Adib et al. also reported that the most common type of cancer in patients with an IVC filter was ovarian cancer (61.5%) [21]. The criteria for insertion of an IVC filter by Adib et al. were (1) surgery was the primary treatment for cancer, (2) surgery was part of definitive treatment, (3) anticoagulation was contraindicated, or (4) after failed anticoagulation [21]. Based on the above results, we perform VTE screening just before surgery in patients with ovarian cancer, a tumor diameter ≥10 cm, or a history of VTE. It is also important to consider use of an IVC filter as required.

### Characteristics of postoperative VTE in patients with gynecologic diseases (Table 6)

Screening for postoperative VTE by interview and measurement of D-dimer identified this condition in 14 out of 1,232 patients (1.14%). These 14 patients consisted of 10 of the 368 patients undergoing cancer surgery (2.72%) and four of the 864 patients having operations for benign diseases (0.46%). Eight of these 14 patients were given both IPC and the anticoagulant danaparoid sodium. The operating time and mean blood loss of the 14 patients were 209.4 (58-375) minutes, and 1001.4 (10-3,230) ml respectively. Santoso et al. reported that the incidence of postoperative DVT was 4.40% (30/684) and 0.30% (2/720) in patients with malignant and benign diseases, respectively [6], while Martino et al. reported that the incidence was 4.1% (21/507) and 0.3% (1/332), respectively [13]. The difference of the incidence after cancer surgery between our results and those of Santoso et al. [6] or Martino et al. [13] may be related to different postoperative prevention methods and differences of patient characteristics such as ethnicity. The fact that we perform screening for preoperative VTE just before surgery might also contribute to a lower incidence of postoperative VTE.

**Table 6:** Risk factors for postoperative VTE

Univariate analysis				
**Variable**		*p *value		

Age ≥50 years		<0.01		
Age ≥55 years		<0.01		
Malignant disease		<0.01		
Operating time ≥4 hours		<0.05		
Blood loss ≥1,000 ml		<0.05		
Blood loss ≥1,500 ml		<0.05		
Blood loss ≥2,000 ml		<0.01		
Blood transfusion ≥2,000 ml		<0.05		

**Multivariate analysis**				

Variable	Risk ratio	95% CI		*p *value
		Lower	Upper	

Allergic-immunologic disease	4.816	1.010	22.956	<0.05
Malignant disease	6.035	1.881	19.359	<0.01
Blood transfusion ≥2,000 ml	6.206	1.248	30.862	<0.05

CI: confidence interval; VTE: venous thromboembolism.

The 10 cancer patients with postoperative VTE consisted of five (3.5%) with ovarian cancer, three (3%) with uterine cervical cancer, and two (1.7%) with endometrial cancer. Unlike preoperative VTE, ovarian cancer was not significantly associated with postoperative VTE. Two of the three patients with cervical cancer had adenocarcinoma and one patient with endometrial cancer had clear cell adenocarcinoma, suggesting the possibility that uterine adenocarcinoma may be associated with a higher risk of postoperative VTE. Two of the five ovarian cancer patients also had clear cell adenocarcinoma. Santoso et al. found no difference in the incidence of postoperative DVT in relation to the type of cancer [6], but Martino et al. reported that the incidence of postoperative PE was 2.8% (2/72) for uterine cervical cancer, 1.2% (2/165) for endometrial cancer, and 6.8% (16/237) for ovarian cancer [13]. Because of the strong possibility that VTE is present preoperatively in ovarian cancer patients, Martino et al. suggested that exclusion should be performed by preoperative screening in patients with suspected ovarian cancer.

The mean age, mean BMI, mean tumor diameter, mean operating time, and mean blood loss of the 10 cancer patients with postoperative VTE was 60 (40-75) years, 21.9 (16.4-27.8) kg/m^2^, 9 (3-20) cm, 235.5 (58-375) minutes, and 1,139 (10-3,230) ml. However, four patients with benign disease included ovarian tumors (n = 2), uterine myoma (n = 1), and intrauterine infection (n = 1). The mean age, mean BMI, mean tumor diameter, mean operating time, and mean amount of blood loss in these four patients were 53.8 (44-64) years, 23.0 (19.6-27.2) kg/m^2^, 12 (6-23) cm, 144.3 (75-176) minutes, and 1,154 (30-2,100) ml, respectively. There were no significant differences of these parameters between the 10 cancer patients and four patients with benign disease. Five patients had DVT only, three had PE only, and PE was combined with DVT in six patients.

### Factors related to postoperative VTE

Univariate analysis using 21 variables showed that age (≥50 or ≥55 years), a diagnosis of cancer, an operating time ≥4 hours, blood loss (≥1,000 ml, 1,500 m, or 2,000 ml), and blood transfusion ≥2,000 ml were significantly related to postoperative VTE (Table 6). When multivariate analysis was performed using 13 variables, significant associations were detected with cancer surgery tumors, a history of allergic-immunologic disease, and blood transfusion ≥2,000 ml, with risk ratios of 6.035, 4.816, and 6.206, respectively. Our previous multivariate analysis of 6,218 patients in whom only IPC was used for prevention of VTE showed that cancer surgery, BMI ≥25 kg/m^2^, and blood transfusion were independent risk factors for postoperative PE [4]. The fact that many of the 1,232 patients in the present series received both IPC and anticoagulation may be one of the reasons why a BMI ≥25 kg/m^2 ^was not identified as a significant risk factor. However, Martino et al. only used IPC for prevention and identified cancer surgery and an age ≥60 years as risk factors for postoperative PE [13]. A recent report indicated that the D-dimer level on postoperative day 3, use of recombinant human erythropoietin, and blood group (non-O) were independent risk factors for postoperative VTE in Japanese patients with gynecologic cancer [22].

## Conclusions

In patients with perioperative VTE, PE often leads to fatal sequelae, so adequate screening for perioperative VTE should be performed in the gynecologic field as is done for orthopedic surgery. VTE should be prevented by careful assessment of patients with the risk factors identified in this study. Because the presence of VTE before the start of treatment is a strong possibility in ovarian cancer patients, preoperative screening is important and consideration should be given to insertion of an IVC filter when required.

## Competing interests

The authors declare that they have no competing interests.

## Authors' contributions

NS and YK were involved in the sequence alignment and drafted the manuscript.

NS and NY(Yoshioka) were involved in writing of method.

NS and TO were involved in writing manuscript.

NS, NY(Yoshioka), TO, TN, NY(Yahagi) and SI were involved in analysis of data.

NS, NY(Yoshioka), MY, KT, YN, KK, and BI were involved in the design of the study.

NS and SI performed the statistical analysis.

YK and NY(Yoshioka) helped to draft the manuscript.

NS, MY, KT and KK were involved in planning, experimental setup.

All authors read and approved the final manuscript.
